# Targeting NEK Kinases in Gastrointestinal Cancers: Insights into Gene Expression, Function, and Inhibitors

**DOI:** 10.3390/ijms26051992

**Published:** 2025-02-25

**Authors:** Lei Chen, Heng Lu, Farah Ballout, Wael El-Rifai, Zheng Chen, Ravindran Caspa Gokulan, Oliver Gene McDonald, Dunfa Peng

**Affiliations:** 1 Department of Surgery, Miller School of Medicine, University of Miami, Miami, FL 33136, USA; lxc1148@miami.edu (L.C.); hxl697@miami.edu (H.L.); fxb414@miami.edu (F.B.); wxe45@miami.edu (W.E.-R.); zxc322@miami.edu (Z.C.); rxc988@miami.edu (R.C.G.); 2 Sylvester Comprehensive Cancer Center, University of Miami, Miami, FL 33136, USA; ogm443@miami.edu; 3 Department of Pathology, Miller School of Medicine, University of Miami, Miami, FL 33136, USA

**Keywords:** gastrointestinal (GI) cancers, NEK, gene expression, therapeutic targets, inhibitors

## Abstract

Gastrointestinal (GI) cancers, which mainly include malignancies of the esophagus, stomach, intestine, pancreas, liver, gallbladder, and bile duct, pose a significant global health burden. Unfortunately, the prognosis for most GI cancers remains poor, particularly in advanced stages. Current treatment options, including targeted and immunotherapies, are less effective compared to those for other cancer types, highlighting an urgent need for novel molecular targets. NEK (NIMA related kinase) kinases are a group of serine/threonine kinases (NEK1-NEK11) that play a role in regulating cell cycle, mitosis, and various physiological processes. Recent studies suggest that several NEK members are overexpressed in human cancers, including gastrointestinal (GI) cancers, which can contribute to tumor progression and drug resistance. Among these, NEK2 stands out for its consistent overexpression in all types of GI cancer. Targeting NEK2 with specific inhibitors has shown promising results in preclinical studies, particularly for gastric and pancreatic cancers. The development and clinical evaluation of NEK2 inhibitors in human cancers have emerged as a promising therapeutic strategy. Specifically, an NEK2 inhibitor, T-1101 tosylate, is currently undergoing clinical trials. This review will focus on the gene expression and functional roles of NEKs in GI cancers, as well as the progress in developing NEK inhibitors.

## 1. Introduction of GI Cancers

Gastrointestinal (GI) cancers refer to a group of cancers that affect the digestive system, including the esophagus, stomach, intestine, pancreas, liver, bile duct, and gallbladder. GI cancers represent a significant global burden, ranking within the top 10 in both incidence and mortality among all cancers [1,2]. According to the Global Cancer Statistics from 2022 [2], esophageal cancer ranks 11th in incidence and 7th in mortality. Gastric cancer ranks fifth in incidence and fifth in mortality. Colorectal cancer ranks third in incidence and second in mortality. Pancreatic cancer ranks 12th in incidence and 6th in mortality. Liver cancer ranks sixth in incidence and third in mortality, while gallbladder cancer ranks 22nd in incidence and 20th in mortality. While the incidence of gastric cancer [3] is declining, the incidence rates of esophageal cancer [4,5], particularly esophageal adenocarcinoma [6], as well as colorectal cancer [7], liver cancer [8,9], and pancreatic cancer [10], are rising globally. Unfortunately, the overall prognosis for patients with GI cancers remains dismal: the 5-year overall survival rate for esophageal cancer is about 20% [11]; for pancreatic cancer, it is about 10% [12]; for gastric cancer, it is about 35% [13]; for liver cancer, it is about 55% [14]; and for colorectal cancer, it is about 64% [15]. The mortality rates vary broadly depending on the stage of diagnosis and geographic region. Although there has been significant improvement in survival for early cancers, the survival rates of most GI cancers have not significantly improved for advanced stages and metastatic cancers. Therefore, there is an urgent need to identify novel potential molecular targets and therapeutic strategies for GI cancers. Conventional therapies for GI cancers include surgery, chemotherapy (such as docetaxel, oxaliplatin, 5-fluorouracil, leucovorin, and capecitabine), and radiotherapy [16]. However, these treatments are often toxic and can cause numerous unwanted side effects, including an increased risk of developing second tumors in long-term survivors. In recent years, significant advances have been made in novel therapeutic strategies for GI cancer [17,18]. These include immunotherapies (such as anti-PD1-PD-L1 and CTLA-4 inhibitors and chimeric antigen receptor T-cell (CART) therapy), targeted therapies (such as antibody drug conjugation (ADC)-like T-DXd for HER2-positive cancers [19], erlotinib for EGFR-positive cancers [20], and many others [18]), RNA therapy [21], and gene therapy and their combinations. Despite these advancements, only a small proportion of patients with GI cancer respond to these novel therapies. Significant challenges remain, and further efforts are needed to identify novel molecular targets for GI cancers.

## 2. Introduction of NEK Kinase Family

**Figure 1:**
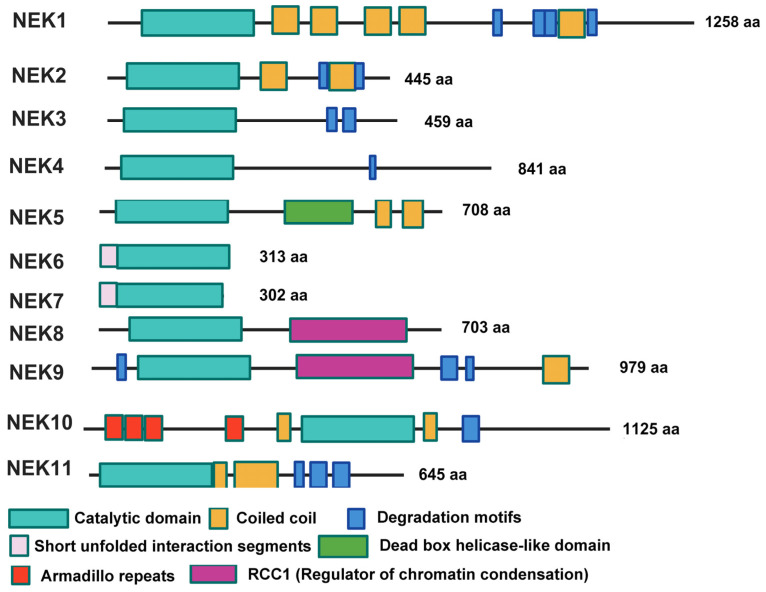
A cartoon showing the protein domain structure of members of the NEK kinase family.

NEK represents NIMA (never in mitosis gene A)-related kinase. NIMA (never in mitosis gene A) kinase was initially identified in aspergillus as a protein responsible for regulating mitotic progression [22,23]. The NIMA family of serine/threonine kinases contains a diverse group of protein kinases involved in various cellular processes, including cilia regulation, microtubule dynamics, mitotic processes, cell growth, and DNA damage response. In humans, 11 members of the NEK family, NEK1-NEK11, are named in the order of their discovery [23,24,25]. All NEKs share a conserved catalytic kinase domain located in the N-terminal of NEKs except for NEK10, which defines them as NIMA-related kinases (Figure 1). These catalytic domains contain all the motifs that are typical of a serine/threonine kinase and share closer sequence similarity with NIMA in aspergillus. The catalytic domain of NEK10 is in the middle of its sequence, making it an exception to other NEKs. The C-terminals of NEKs are quite different in their structures. Most of them (NEK1, NEK2, NEK5, NEK9, NEK10, and NEK11) contain one or several coiled coil domains, which promote autophosphorylation and activation. NEK8 and NEK9 have a regulator of chromatin condensation (RCC1) domain, which connects them to the RCC1 superfamily. Interestingly, NEK6 and NEK7 lack a C-terminal domain, primarily consisting of mainly a kinase domain with a short N-terminal extension, known as a short, unfolded interaction segment. Functionally, NEK protein kinases are primarily serine/threonine kinases, except for NEK1 and NEK10, which exhibit dual-kinase activities involving both serine/threonine and tyrosine [26,27]. Under normal physiological conditions, most NEKs play a crucial role in regulating cell cycle progression [23,28], particularly during mitosis [29]. NEK1, NEK10, and NEK11 are involved in regulating the DNA damage response (DDR) [30]. Additionally, some NEKs, such as NEK1 and NEK8, are involved in cilium biogenesis [31], while others, including NEK1, NEK4, NEK5, NEK6, and NEK10, regulate mitochondrial function [32]. NEK2 has also been reported to play a role in meiosis [33] and alternative RNA splicing [34,35]. NEK7 is specifically involved in the regulation of NLRP3 inflammasome activation [36], which is associated with many diseases including cancer (Table 1).

## 3. Gene Expression, Functions, and Molecular Mechanisms of NEK Kinase Family Members in GI Cancers

### 3.1. Esophageal Cancer

The NEK2 mRNA and protein levels are upregulated in esophageal squamous cell carcinoma (ESCC) [37,38]. The overexpression of NEK2 in ESCC promotes tumor cell migration, invasion, and proliferation and contributes to the radio resistance of ESCC cells [39]. There are limited data available on the gene expression of the NEKs in esophageal adenocarcinoma (EAC). We have reported that the gene expression levels of NEK2, NEK3, and NEK6 are frequently upregulated in esophageal adenocarcinoma as compared to normal esophageal epithelium [40]. Among these genes, NEK2 is the most frequently upregulated NEK in esophageal adenocarcinoma at both the mRNA and protein levels [40]. The gene expression and potential functions of other NEK members in esophageal cancer remain largely unknown.

### 3.2. Gastric Cancer

Both the mRNA and protein levels of NEK2 are significantly upregulated in gastric cancer (GC) as compared to normal stomach samples [41,42,43]. The overexpression of NEK2 in GC promotes tumor progression by activating AKT signaling and metabolism [41] and also by regulating ERK/MARK signaling [42]. It is also reported that NEK2 promotes GC proliferation, migration, and growth by regulating the KDM5B/H3K4me3 axis [44]. A high level of NEK2 affects ferroptosis sensitivity through the Keap1/Nrf2/HMOX1 axis [45]. Additionally, a high expression of NEK2 may contribute to a suppressive tumor immune microenvironment [43] and is associated with a poor prognosis for patients with GC [43]. Cao and colleagues [46] reported that the NEK3 mRNA and protein levels were overexpressed in GC compared to adjacent normal tissues. This overexpression was found to be associated with advanced stages of the disease and a poor prognosis [46]. NEK6 was identified as one of the top six upregulated genes in GC in integrated gene expression profiling and network analyses [47], which was validated using Western blotting and immunohistochemistry. It suggested that NEK6 may play a role in GC tumorigenesis and progression. Dr. Li and colleagues [48] reported that NEK7 is upregulated in GC samples compared to normal samples, and a high level of NEK7 is associated with an advanced T stage and poor prognosis. Silencing NEK7 confirmed its role in promoting GC tumor growth both in vitro and in vivo xenograft models. Regarding NEK8 in GC, Ding and colleagues [49] reported that NEK8 promotes GC cell proliferation and migration and is associated with a poor prognosis. They also identified that the von-Hippel–Lindau tumor suppressor protein (pVHL) regulates the protein stability and proteosome degradation of NEK8. In two publications, Lu and colleagues [50,51] investigated the role of NEK9 in GC. They found that both the mRNA and protein levels of NEK9 are elevated in GC compared to normal samples. The high expression of NEK9 is associated with tumor cell motility and metastasis. Researchers have also identified NEK9 as the downstream effector of IL-6/STAT3. There have been no publications available for NEK1, NEK4, NEK5, NEK10, and NEK11 in GC thus far.

### 3.3. Colorectal Cancer (CRC)

NEK2 is overexpressed in colorectal cancers compared to normal samples. The overexpression of NEK2 is associated with tumor invasion, advanced stages, and a poor prognosis of CRC [52,53,54]. Targeting NEK2 may enhance tumor cell sensitivity to cisplatin in colon cancers [55]. Mechanistically, the overexpression of NEK2 in colorectal cancers may be regulated by upstream miRNA-128 methylation [54] and FBXW7 circle RNA [56]. NEK4 has been identified as an aberrant overexpressed gene and may contribute to TRAIL resistance as the inhibition of NEK4 sensitizes CRC cells to TRAIL-induced cell death. Dr. Korkmaz’s [57,58] laboratory investigated the gene expression of NEK6 in CRC. They found that NEK6 expression, together with AURKA and PAK1, is upregulated in precancerous uncreative colitis [57] and adenomatous polyps [58] and remains high in CRC samples. This indicates an early event that may serve as a biomarker for predicting CRC. Cai and colleagues [59] reported that both the mRNA and protein levels of NEK8 were upregulated in CRC, and NEK8 regulates CRC progression by phosphorylating the oncogene C-MYC at s405. Kim and colleagues explored the role of NEK9 in CRC and found that the overexpression of the NEK9-EG5 axis is associated with the distal metastasis of CRC, suggesting that it may serve as a biomarker [60]. Sabir and colleagues [61] demonstrated that NEK11 is necessary for the G2/M cell cycle arrest of CRC cells induced by therapeutic DNA damage agents. As of now, there are no publications available for NEK1, NEK3, NEK7, or NEK10 in CRC.

### 3.4. Pancreatic Cancer

Pancreatic ductal adenocarcinoma (PDAC) is the deadest human malignancy with a 5-year overall survival rate of about 10%. The NEK2 gene has been identified as one of the ten hub genes strongly associated with the progression of PDAC [62]. Recently, Zhang and colleagues [63] reported that both the mRNA and protein levels of NEK2 are significantly upregulated in PDAC. The high NEK2 level in PDAC is associated with poor prognosis, particularly in immune-hot tumors. They also found that NEK2 phosphorylates PD-L1 at T194/T210 to render the tumor cells resistant to immune therapy. Additionally, NEK6 has been reported to enhance the ubiquitination and degradation of USP49 by FBXO45 in pancreatic cancer cells [64]. However, the gene expression of NEK6 in PDAC remains unclear. Both the mRNA and protein levels of NEK7 are upregulated in PDAC as compared to normal pancreas tissues [65]. A high level of NEK7 promotes PDAC cell proliferation, migration, and invasion and is associated with advanced tumor stages, liver metastasis, and poor prognosis [65]. Nie and colleagues reported that NEK9 may be involved in the regulation of the Hippo pathway in pancreatic cancer [66].

### 3.5. Liver Cancer

NEK2 has been well studied in liver cancer, with more than 30 publications on the subject. Most of these publications [67,68,69,70] have reported that NEK2 is overexpressed in hepatocellular carcinomas (HCCs). Elevated NEK2 levels have been associated with tumor proliferation [67], migration, invasion, EMT [68], stemness [71], recurrence [72], drug-resistance [70], and poor prognosis [69,71]. However, one publication [73] claimed that low NEK2 expression levels were associated with poor prognosis. Mechanistically, NEK2 has been shown to activate the Wnt/β-catenin pathway to promote tumor progression [74] and drug resistance [70,75]. A high level of NEK6 is reported in HCC compared to adjacent normal tissues. This is associated with higher proliferation (Ki67), elevated Alpha-Fetoprotein (AFP) levels, and poor prognosis [76]. Zuo and colleagues [77] reported that NEK6 interacts with Smad4 and may negatively regulate TGFβ signaling in HCC cells. Additionally, Zhou and colleagues [78] reported that both the mRNA and protein levels of NEK7 are overexpressed in HCC. The high level of NEK7 was significantly correlated with advanced stages, high proliferation (Ki67) rates, and poor prognosis.

### 3.6. Gallbladder Cancer (GBC) and Cholangiocarcinoma

Limited studies are available about NEK family members in gallbladder cancer and cholangiocarcinoma. One in silico analysis identified NEK2 as one of the three hub genes (TRIP13, NEK2, and TPX2) associated with GBC mortality [79]. Wang and colleagues [80] analyzed the gene expression of NEK7, FOXM1, and PIK1 in GBC and found that a high NEK7 expression level is associated with a poor prognosis for patients with GBC. Kokuryu and colleagues [81] reported that NEK2 is an effective target for inhibiting tumorigenic growth and the peritoneal dissemination of cholangiocarcinoma.

**Figure 2:**
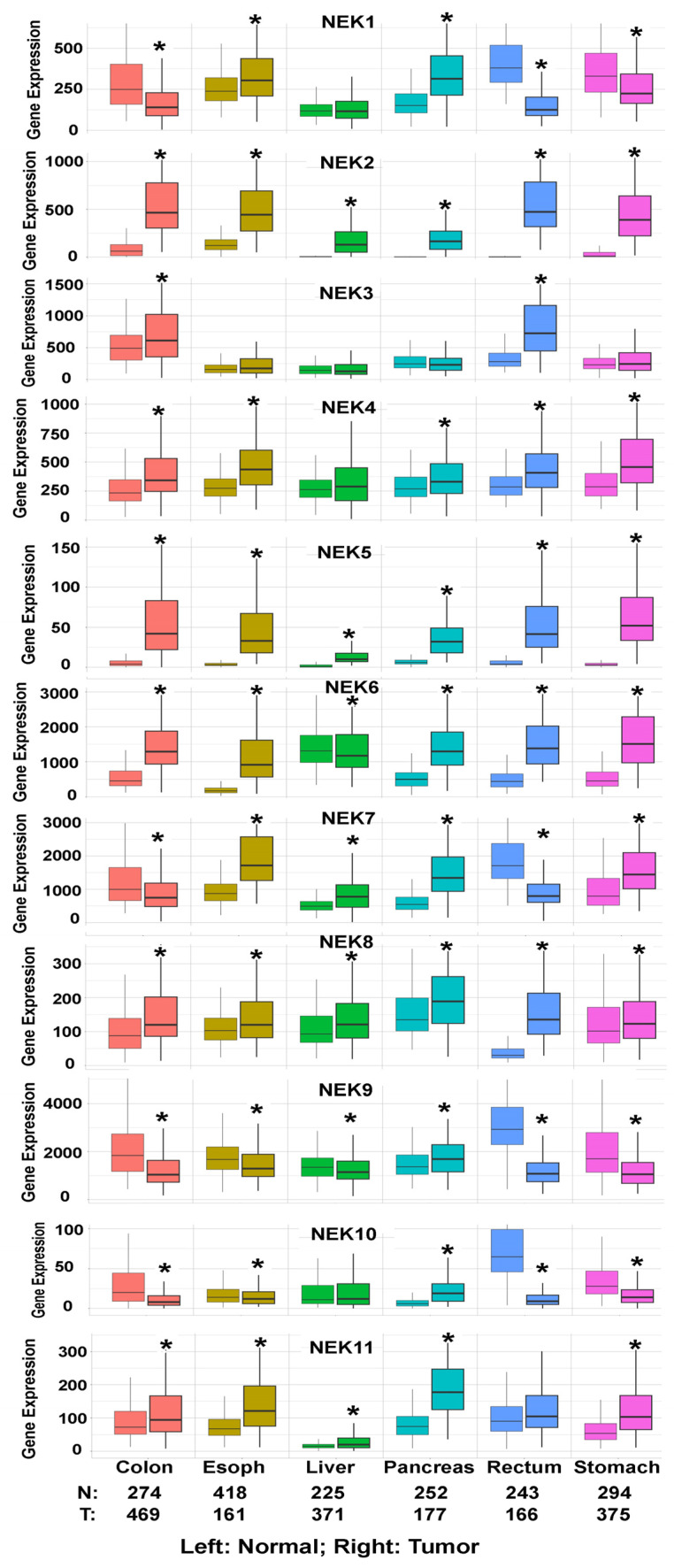
The gene expression of NEK family members in GI cancers. The data are from TNMplot databases of colon, esophagus (Esoph), liver, pancreas, rectum, and stomach, including normal and tumor samples. The comparison of the gene expression of the NEKs between tumor and normal samples was performed using the online TNMplot tools. * *p* < 0.05. The different colors are used to differentiate the tumor types; one color represents one type of GI cancer.

In addition to the above literature available on NEKs in GI cancers, we conducted a bioinformatics analysis on the gene expression of NEK1-NEK11 in GI cancers using online TNMplot databases (https://tnmplot.com/analysis/, accessed on 30 November 2024). These databases include 56,938 unique samples from GEO, GTex, TCGA, and TARGET databases [82]. Unfortunately, the dataset does not include data for gallbladder cancer. The results are shown in Figure 2. The results confirm the upregulation of NEK2, NEK4, NEK5, NEK6, NEK8, and NEK11 in most GI cancers, while the level of NEK9 is downregulated in most GI cancers. It is worth noting that the baseline gene expression of some NEKs, such as NEK5, NEK8, and NEK10, are quite low. Further validation is needed to determine the pathophysiological effect of the changes shown here.

## 4. Are NEK Kinase Family Members Good Targets for GI Cancers?

### 4.1. Targeting NEK2 in GI Cancer

From the above review of the literature, NEK2 is the most studied NEK members in GI cancers and in other cancers [83,84,85]. The overexpression of NEK2 is observed in all the GI cancers and is associated with tumor proliferation [38,44,86], invasion [44,86], drug resistance [75,87], and poor survival [86,88]. By conducting a combined integrative and bioinformatic analysis of the Clinical Proteomic Tumor Analysis Consortium (CPTAC) datasets, Deb and colleagues [89] discovered that NEK2 and Aurora kinase A are the most activated kinases among the five common human cancers (ovarian cancer, breast cancer, colon cancer, lung adenocarcinoma, and endometrial cancer). Therefore, targeting NEK2 represents a promising therapeutic approach on its own or in combination with other therapeutic strategies [63]. Significant efforts have been made to develop specific NEK2 inhibitors, with several potent candidates showing encouraging outcomes in both in vitro and in vivo models [90,91,92,93,94,95]. Among these, T-1101 tosylate [95] has entered phase I clinical trials, exhibiting promising anti-tumor efficacy across various types of cancer.

#### 4.1.1. SU11652

SU11652 is the first reported NEK2 inhibitor. Originally designed to target tyrosine kinases, it was later found to inhibit several serine/threonine protein kinases. SU11652 is a cell-permeable compound that acts as a potent ATP-competitive tyrosine kinase receptor. It inhibits the activities of vascular endothelial growth factor receptor 2 (VEGFR2), platelet-derived growth factor receptor (PDGFR), fibroblast growth factor receptor (FGFR), epidermal growth factor receptor (EGFR), and Kit family members with a broad range of IC50 values ranging from 3 nM to 20 µM [96,97]. Rello and colleagues [97] found that SU11652 binds to NEK2 and shows modest NEK2 inhibitory activities, with an IC50 value of 8 µM determined by the peptide substrate of GTFRSSIRRLSTRRRY (Km ~90 µM, kcat ~17 min^−1^). Following this research, a series of aminopyrazines [98,99] were designed to target NEK2. However, none of the aminopyrazines are active in cells, possibly due to insufficient membrane permeability.

#### 4.1.2. NCL 00017509 (NEK2-IN-5)

NCL 00017509 [100] is a potent and selective NEK2 inhibitor that targets the enzyme’s cysteine 22 residue near the catalytic site. Dr. Zhang and colleagues [63] administered NCL 00017509 to C57BL/6 mice and nude mice with pancreatic cancer to evaluate its therapeutic effects. They found that NCL 00017509 significantly suppressed tumor growth in tumor-bearing C57BL/6 mice but not in tumor-bearing nude mice. They showed that NCL 00017509 reduces PD-L1 levels by inhibiting NEK2, which enhances T cell function in the tumor microenvironment and sensitizes tumor cells to immune therapy.

#### 4.1.3. JH295

JH295 is the first irreversible NEK2 inhibitor tested in cells. Henise and colleagues [101] used a structure-based strategy to design irreversible cysteine-targeted inhibitors of NEK2. They discovered that oxindole propynamide 16 (JH295) is a potent NEK2 inhibitor in biochemical and cell-based assays. Of note, JH295 irreversibly and selectively inhibits NEK2 activity without affecting the activities of other mitotic kinases, such as CDK1, Aurora B, PIK1, and MPS1. JH295 induced tumor cell apoptosis and promoted chemosensitivity to other chemotherapeutic drugs in lymphoma cells [102]. However, there have been no publications reporting the use of JH295 in GI cancers or its evaluation in clinical trials.

#### 4.1.4. MBM

Dr. Fang [103] and Dr. Xi [93] and colleagues designed and analyzed a series of imidazo[1,2-*a*]pyridine derivatives of potent NEK2 inhibitors. Dr. Fang and colleagues demonstrated that MBM-5 occupies the ATP-binding site and forms three conserved hydrogen bonds with NEK2; one is between the nitrogen atom of imidazole[1,2-a]pyridine and CYS 89, and the other two are between the amide group and ASP 159 [103]. The inhibitory efficacy of MBM-5 on NEK2 has been evaluated by examining the phosphorylation of HEC1 (S165), a known downstream substrate of NEK2 [104]. The results show that MBM-5 enters cells and inhibits cellular NEK2 kinase activity in a concentration- and time-dependent manner without affecting the phosphorylation of Aurora A [103]. MBM-5 also induces G2/M phase cell cycle arrest, promotes polyploidy, and triggers apoptosis. Additionally, it has been demonstrated that MBM-5 inhibits tumor cell growth in vitro and effectively suppresses tumor growth in human gastric and colon cancer xenografts [103]. Dr. Xi and colleagues reported that MBM-17 and MBM-55 displayed a significant suppression of tumor growth in vivo without apparent toxicity [93]. These MBM compounds are worth further investigation.

#### 4.1.5. NBI-961 (Formerly CMP3a)

NBI-961 is a recently developed NEK2 selective inhibitor [92]. NBI-961 demonstrates bifunctional effects on NEK2 by inhibiting its catalytic activity through the suppression of NEK2 phosphorylation and reducing its protein levels via proteasome-mediated degradation. It induces G2/M arrest and apoptosis in multiple DLBCL (diffuse large B cell lymphoma) cell lines and delays DLBCL tumor growth in vivo [105]. However, NBI-961 has not been assessed in GI cancers or evaluated in clinical trials.

#### 4.1.6. INHs (Inhibitor for Nek2 and Hec1 Binding)

HEC1 is a known NEK2 downstream target, a critical mitotic regulator responsible for spindle checkpoint control, kinetochore functionality, and cell survival [104,106]. The overexpression of HEC1 has been found in various cancer types [107]. INH1 (N-(4-[2,4-dimethyl-phenyl]-thiazol-2-yl)-benzamide) was identified through a chemical genetic screening conducted by Dr. WH Lee’s laboratory [108]. This compound disrupts the HEC1/NEK2 interaction by directly binding to HEC1 and reducing the overall global NEK2 protein level [108]. The preliminary test of INH1 in human cells demonstrated that INH1 inhibits the tumor xenograft growth of a human breast cancer line MDA-MB-468 in a nude mouse model with no apparent side effects. The laboratory later developed a series of INH analogs and evaluated them in human cells. INH154 is a derivative of INH, which binds to HEC1 at amino acids 394–408 on W395, L399, and K400 residues [109]. INH154 was shown to effectively block NEK2-mediated phosphorylation on S165 of HEC1 and kill cancer cells at the nanomolar range both in vitro and in vivo in a xenograft mouse model [109]. However, there are currently no publications available evaluating these INHs in GI cancers or reporting on their testing in clinical trials.

#### 4.1.7. TAI-1

TAI-1 [110] is a specific compound modified from the above INH1 by introducing C-6′ methyl, C-4′ 4-methoxyphenoxy, and 4-pyridyl groups into the core structure of INH1. TAI-1 has a GI_50_ value of 13.48 nM (K562 cells), which represents a nearly 1000-fold increase in potency compared to INH1 (GI_50_  =  11.7 μM). Of note, TAI-1 specifically targets NEK2/HEC1, exhibiting minimal to no effect on a range of other kinases tested, such as CHK1/CHK2, Cdk1/Cyclin B, Aurora A/B, mTOR, PI3K, etc. TAI-1 inhibits cellular growth at the nM level for most cancer cell lines screened, including breast cancer, liver cancer, colon cancer, leukemia, and others. It has been shown to suppress xenograft tumor growth in multiple tumor types with no or minor toxicity [110].

#### 4.1.8. TAI-95/T-1101 Tosylate

Building on TAI-1, the developers (Dr. Huang and colleagues) introduced a series of heterocycles at the C-4′ position and finally developed TAI-95, which has a C-4′ 2-methoxyethoxy thiopyrazine and retains excellent potency (IC_50_ = 22.21 nmol/L in MDA-MB-468 cells). Preliminary tests demonstrated that TAI-95 significantly suppresses breast cancer cell growth in vitro and strongly inhibits in vivo tumor growth when administered orally without causing significant weight loss or other obvious toxicities [111]. Subsequently, the group further developed TAI95 as a first-in-class oral clinical candidate for NEK2/HEC1 inhibition with potential for cancer therapy. This compound is named T-1101 tosylate [95]. T-1101 has good oral absorption and potent antiproliferative activity (IC50: 14.8–21.5 nM) in vitro. It shows promising antitumor efficacy in human tumor xenografts of liver cancer and breast cancer when administered orally. Additionally, T-1101 has shown synergistic anticancer effects when used in combination with doxorubicin, paclitaxel, or topotecan. Currently, T-1101 tosylate is in phase I clinical trials as an orally administered drug for cancer therapy.

**Figure 3:**
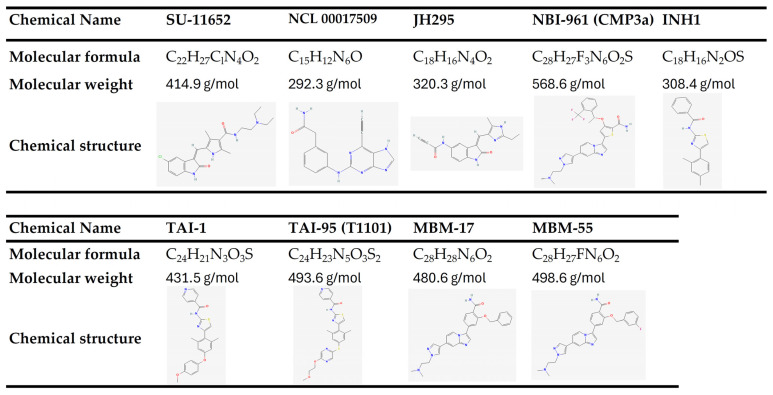
Basic chemical information of NEK2 inhibitors. All information in this figure was retrieved from PubChem database (https://pubchem.ncbi.nlm.nih.gov, PubChem) on 19 February 2025.

Basic chemical information, including the chemical structure of these NEK2 inhibitors, is shown in Figure 3.

In summary, targeting NEK2 using specific inhibitors exhibits promising antitumor efficacy in GI cancers. In particular, when combined with other therapeutic strategies, NEK2 inhibition sensitizes tumor cells to traditional chemotherapy and radiotherapy, making it a promising strategy for cancer therapy by integrating chemotherapy, targeted therapy, and immunotherapy [83]. More efforts are needed to evaluate NEK2 inhibitors in clinically relevant models of GI cancers.

### 4.2. Targeting Other NEKs in Cancers

#### 4.2.1. Targeting NEK6

In addition to NEK2, which is the most overexpressed NEK in human cancers, including GI cancers, NEK6 overexpression has been reported in various human cancers, making it an attractive target for antitumor therapy [112,113,114]. Donato and colleagues [115] employed in silico screening techniques to search for novel NEK6 inhibitors and identified compound 8, which shows selective inhibition of NEK6 and NEK1 with no effect on NEK7. In a preliminary test, compound 8 displayed antiproliferative activity against a panel of human cancer cell lines and showed a synergistic effect with cisplatin or paclitaxel in a BRCA2 mutated ovarian cancer cell line. Further refinement through structure–activity relationship (SAR) studies is necessary to enhance its activity and enable future applications.

#### 4.2.2. Targeting NEK7

NEK7 overexpression has been detected in various human cancers, including GI cancers [48,65,78], where it plays a role in promoting tumor progression. Targeting NEK7 has emerged as a promising therapeutic approach for diseases involving NEK7 dysfunction, such as NLRP3-related diseases and cancers [116,117]. Dr. Aziz and colleagues [118,119,120] employed several strategies to identify specific NEK7 inhibitors. They recently utilized deep learning and structure-based virtual screening of 1200 benzene sulphonamide derivatives retrieved from the PubChem database. They validated their findings through docking studies with the crystal structure of the NEK7 protein [120]. This process led to identifying compound 762 as a potential NEK7 inhibitor. Dr. Ejaz and colleagues [121] conducted comprehensive computational analyses of the PubChem database and FDA-approved protein kinase inhibitors. They identified Alectinib, Crizotinib, and compound 146476703 as potent NEK7 inhibitors. Additionally, using in silico analysis, Dr. Adrees and colleagues [122] identified boeravinone B as a compound that targets NEK7 and PPP1CA proteins, which are overexpressed in pancreatic ductal adenocarcinoma, suggesting that targeting NEK7 may be a promising approach for pancreatic cancer. However, most of these compounds have not yet been evaluated in human cell models or in vivo animal models.

## 5. Summary and Perspectives

NEK kinase family members are relatively novel, less studied druggable targets compared to some well-known kinases like Aurora kinases, CDKs, and others. Among the 11 NEKs, NEK2 is the most extensively studied in human cancers. NEK2 overexpression is observed in various cancers, including gastrointestinal (GI) cancers, and is associated with tumor proliferation, progression, drug resistance, and poor prognosis. Consequently, significant efforts have been made to develop NEK2 inhibitors. Some potent inhibitors have shown promising antitumor activity in human cell models and in vivo mouse models. Notably, T-1101 tosylate is currently undergoing clinical trials, showing promising antitumor efficacy with minimal toxicity. Due to the high frequency of NEK2 overexpression in GI cancers, the development and evaluation of novel NEK2 inhibitors for clinical applications in GI cancers are both promising and necessary.

Further research is required to understand the roles of other NEKs in the development and progression of GI cancers. The overexpression of NEK6 and NEK7 has been observed in several GI cancers, warranting additional investigation. While some publications have reported the development of NEK6 and NEK7 inhibitors, these are still in the preliminary stages of drug development. Additional efforts are needed to develop and evaluate potent inhibitors for other NEKs.

## Figures and Tables

**Table 1:** Summary of basic information for NEK family members.

Gene	Chromosome Localization ^#^	Cytoband *	Protein Size *	Subcellular Location *	Molecular Function *	Biological Process *
*NEK1*	chr4:169392809-169612583	4q33	1258 aa	Cytosol, Nucleoplasm	Serine/threonine-protein kinase, transferase, tyrosine-protein kinase	Cell cycle, cilium biogenesis, mitosis, DDR
*NEK2*	chr1:211662772-211675621	1q32.3	445 aa	Nucleoplasm, Centrosome	Serine/threonine-protein kinase, transferase	Cell cycle, cell division, chromosome partition, meiosis, mitosis, alternative splicing
*NEK3*	chr13:52132647-52159597	13q14.3	459 aa	Cytoplasm, Microtubules	Serine/threonine-protein kinase, transferase	Cell cycle, cell division, mitosis
*NEK4*	chr3:52708444-52770940	3p21.1	841 aa	Cytosol	Serine/threonine-protein kinase, transferase	Cell cycle, cell division, mitosis
*NEK5*	chr13:52033611-52129073	13q14.3	889 aa	Nucleoplasm, Cytosol	Serine/threonine-protein kinase, transferase	Cell cycle, mitochondrial
*NEK6*	chr9:124257949-124353307	9q33.3	313 aa	Nucleoplasm, Cytosol	Serine/threonine-protein kinase, transferase	apoptosis, cell cycle, cell division, chromosome partition, mitosis
*NEK7*	chr1:198156998-198322420	1q31.3	302 aa	Nucleoplasm, Cytosol	Serine/threonine-protein kinase, transferase	Mitosis, NLRP3 inflammasome activation
*NEK8*	chr17:28728788-28743455	17q11.2	703 aa	Cytoplasm, Microtubule, Mitotic Spindle	Serine/threonine-protein kinase, transferase	Cell cycle, ciliary biogenesis, organogenesis, Hippo signaling
*NEK9*	chr14:75079353-75127048	14q24.3	979 aa	Cytoplasm	Serine/threonine-protein kinase, transferase	Cell cycle, cell division, mitosis
*NEK10*	chr3:27106484-27369383	3p24.1	1125 aa	Nucleoplasm, Vesicles	Serine/threonine-protein kinase, transferase, tyrosine-protein kinase	Cell cycle, radiation response, DDR
*NEK11*	chr3:131026877-131350465	3q22.1	645 aa	Nucleoplasm	Serine/threonine-protein kinase, transferase	Cell cycle, DDR

^#^ The chromosome localization is based on the information of the representative transcript from RefSeq and GENCODE. * The information is from The Human Protein Atlas (https://www.proteinatlas.org/, accessed on 10 December 2024).

## Abbreviations

GI cancer	gastrointestinal (GI) cancer
NEK	NIMA (never in mitosis gene A)-related kinase
DDR	DNA damage response
RCC	regulator of chromatin condensation
ESCC	esophageal squamous cell carcinoma
EAC	esophageal adenocarcinoma
GC	gastric cancer
CRC	colorectal cancer
PDAC	pancreatic ductal adenocarcinoma
HCC	hepatocellular carcinomas
AFP	alpha-fetoprotein
GBC	gallbladder cancer
GEO	gene expression omnibus
TCGA	The Cancer Genome Atlas
CPTAC	Clinical Proteomic Tumor Analysis Consortium
VEGFR2	vascular endothelial growth factor receptor 2
PDGFR	platelet-derived growth factor receptor
FGFR	fibroblast growth factor receptor
EGFR	epidermal growth factor receptor
DLBCL	diffuse large B cell lymphoma
FDA	Food and Drug Administration
CDKs	cyclin-dependent kinases
